# Ablação Por Cateter Sem Uso de Fluoroscopia Para Tratamento de Fibrilação Atrial e Arritmias Atriais: Eficácia e Segurança

**DOI:** 10.36660/abc.20200096

**Published:** 2020-06-29

**Authors:** Eduardo B. Saad, Charles Slater, Luiz Antonio Oliveira Inácio, Gustavo Vignoli dos Santos, Lucas Carvalho Dias, Luiz Eduardo Montenegro Camanho

**Affiliations:** 1 Hospital Pró-Cardíaco Serviço de Arritmias e Estimulação Cardíaca Artificial Rio de JaneiroRJ Brasil Hospital Pró-Cardíaco - Serviço de Arritmias e Estimulação Cardíaca Artificial, Rio de Janeiro, RJ - Brasil

**Keywords:** Arritmias Cardíacas, Fibrilação Atrial, Ablação por Cateter, Fluoroscopia, Radiação, Eficácia, Segurança

## Abstract

**Fundamento:**

O uso da radiação ionizante em procedimentos médicos está associado a riscos significativos à saúde dos pacientes e da equipe de saúde.

**Objetivos:**

Avaliar a segurança e a eficácia aguda da ablação por cateter para tratamento da fibrilação atrial (FA) e arritmias supraventriculares (SVTs), utilizando uma abordagem exclusivamente não fluoroscópica, guiada por eco intracardíaco (ICE) e mapeamento 3D.

**Métodos:**

95 pts (idade média 60 ± 18 anos, 61% do sexo masculino) programados para ablação de FA (69 pts, 45 FA paroxística e 24 FA persistente) ou SVTs (26 pts – 14 reentrada nodal, 6 Wolf-Parkinson-White [WPW], 5 flutter atrial direito [AD], 1 taquicardia atrial). Nove pacientes (9,5%) tinham marcapasso definitivo ou dispositivos de ressincronização com desfibrilador. Dois sistemas de mapeamento eletroanatômico foram utilizados – CARTO (65%) e NAVx (35%), bem como cateteres de ICE disponíveis – Acunav e ViewFlex.

**Resultados:**

O isolamento das veias pulmonares (VPs), bem como todos os outros alvos que precisavam de ablação em ambos os átrios, foram alcançados e adequadamente visualizados. Não foram observados derrames pericárdicos, complicações trombóticas ou outras intercorrências nesta série. Punções transseptais difíceis (19 pacientes – 20%) foram realizadas sem fluoroscopia em todos os casos. Não foi utilizada fluoroscopia de backup, e nenhum vestuário de chumbo foi necessário. Avaliações detalhadas dos marcapassos após o procedimento não mostraram nenhum dano aos eletrodos, deslocamentos ou mudanças de limiar.

**Conclusões:**

Uma estratégia de ablação por cateter sem uso de radiação para FA e outras arritmias atriais é segura e eficaz quando guiada pela utilização adequada do ICE e do mapeamento 3D. Diversos sítios em ambos os átrios podem ser alcançados e adequadamente ablacionados sem a necessidade de fluoroscopia de backup. Não foram observadas complicações. (Arq Bras Cardiol. 2020; 114(6):1015-1026)

## Introdução

A ablação por cateter é atualmente o método mais eficaz para o tratamento da fibrilação atrial (FA),^1 , 2^ flutter atrial e taquicardias supraventriculares (SVTs). É amplamente realizado em vários centros ao redor do mundo, dada a crescente prevalência de FA na população e a modesta resposta a medicamentos antiarrítmicos.

Como acontece com a maioria dos procedimentos cardíacos percutâneos, a fluoroscopia tem sido a modalidade primária de imagem para manipular cateteres no espaço vascular e câmaras cardíacas. No entanto, a radiação ionizante tem múltiplos efeitos deletérios tanto para os pacientes quanto para a equipe de saúde envolvida.^3 - 6^ Esses efeitos são cumulativos, e todos estamos continuamente expostos devido ao uso frequente em métodos de imagem com fins diagnósticos terapêuticos.^7^

Neste cenário, o princípio **ALARA** ( **A** s **L** ow **A** s **R** easonably **A** chieveable) foi proposto para ajustar o uso de radiação ao mínimo necessário para atender aos objetivos.^3^ Nos últimos anos, vários avanços foram obtidos para reduzir a exposição à radiação durante os procedimentos de ablação por cateter, incluindo a redução do tempo de exposição e das doses de fluoroscopia,^8 , 9^ melhores métodos de barreira, e, especialmente, o uso crescente de outras modalidades de imagem não fluoroscópica – sistemas eletroanatômicos 3D (EA) e ecocardiografia intracardíaca (ICE).

Estas ferramentas de redução de fluoroscopia têm sido cada vez mais utilizadas no laboratório de eletrofisiologia ao longo dos anos, de tal forma que se tornou possível usá-las para orientar todo o procedimento de ablação e, assim, evitar o uso de raios-X^10^ inteiramente. Relatadas pela primeira vez há cerca de 10 anos,^11 - 13^ estas técnicas “zero-fluoro” estão ganhando popularidade na comunidade eletrofisiológica, pois podem ser tão seguras e eficazes quanto as orientadas pela fluoroscopi.^14 - 16^

## Objetivos

O objetivo deste estudo foi demonstrar a viabilidade e segurança da ablação por cateter para tratamento da FA, flutter atrial e outras taquicardias supraventriculares, sem o uso de fluoroscopia, utilizando exclusivamente o mapeamento eletroanatômico e o ecocardiograma intracardíaco ecocardiografia, em uma série de 95 pacientes consecutivos.

## Métodos

### Descrição da técnica

Todos os procedimentos foram realizados sob anestesia geral e os acessos venosos profundos foram guiados por ultrassom, de acordo com a necessidade específica do procedimento; usualmente, consistiu em duas ou três punções na veia femoral direita, uma punção na veia femoral esquerda (para o cateter ICE), e uma na veia jugular punção interna (em casos de fibrilação atrial, para colocação de cateter duodecapolar no seio coronário [SC]). O monitoramento durante o procedimento incluiu eletrocardiograma de 12 derivações e adesivos cutâneos de mapeamento EA (Ensite NavX – St. Jude Medical, St. Paul, MN, USA ou CARTO 3 – Biosense Webster Inc., Diamond Bar, CA, USA)

### Navegação através do espaço intravascular

A partir da veia femoral esquerda, um cateter ICE (ViewFlex Xtra – Abbott ou Acunav – Biosense Webster) foi avançado para o átrio direito (RA), guiado pela visualização de “espaços livres de eco” no sistema vascular.

O cateter ICE foi avançado através da veia ilíaca esquerda, mantendo um “espaço livre de eco” próximo do campo proximal de imagem do ultrassom (representando uma ausência de contato tecidual na ponta do cateter ICE). Essa técnica permite ao operador discriminar entre o avanço livre através do lúmen vascular e uma resistência ao avanço pela parede vascular quando esse padrão de imagem não é obtido.

Quando o caminho até a veia cava inferior não era tão claro ou tortuoso, a técnica de seguir um fio-guia no lúmen vascular, usando guias longas inseridas através da veia femoral esquerda, permitiu avançar o cateter ICE seguindo a imagem da guia no lúmen. É de particular valor em pacientes com veias ilíacas finas, onde o “espaço livre de eco” pode não ser muito claro.

**Figura 1:**
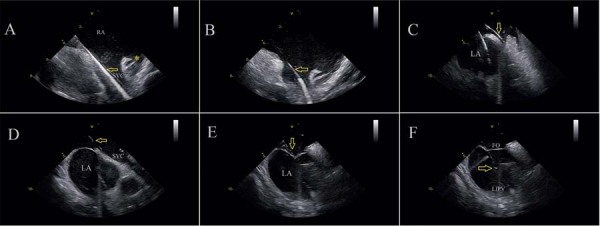
– Sequência de imagens do ICE mostrando os passos para a realização das duas punções transseptais “zero-fluoro”. A) Um fio-guia (seta) é avançado até a veia cava superior (SVC); na imagem, o átrio direito (RA) também é visualizado, bem como o apêndice atrial direito (*), confirmando o posicionamento correto da guia. B) Uma longa bainha transseptal (seta) é avançada sobre a guia para a veia cava superior, apagando o brilho da guia à medida que é avançada. C) O conjunto bainha transseptal com a agulha (seta) na cava superior será, subsequentemente, recuado até a fossa oval. O átrio esquerdo (LA) é visualizado, bem como outro acesso transseptal que já foi realizado previamente. D) Bainha + agulha (seta) sendo recuada ao longo do septo em seu caminho para a fossa oval. E) Bainha + agulha fazendo tenda na fossa oval (seta), confirmando posicionamento adequado para perfurar o septo e acessar o átrio esquerdo (LA).F) Punção da fossa oval (FO) e posicionamento da agulha visualizado na cavidade atrial esquerda (seta). A punção transseptal é realizada na porção posterior do septo, confirmada pela visualização da veia pulmonar inferior esquerda (LIPV) no plano do feixe de ultrassom.

Ao chegar à veia cava inferior, é possível apreciar a junção cavo-atrial onde, ao nível do fígado, uma imagem parenquimatosa com as veias intra-hepáticas é visualizada. Neste momento, é essencial identificar e evitar a progressão inadvertida do cateter ICE através das veias hepáticas e direcioná-lo corretamente para o átrio direito (AD), o que pode ser feito com suave deflexão anterior. Uma vez no AD, todas as visualizações “padrão” podem ser obtidas usando a técnica convencional de torque no sentido horário ou anti-horário a partir da “home view”. O cateter é então prolapsado para ventrículo direito usando deflexão anterior, mantendo a válvula tricúspide visível, e imagens do espaço pericárdico são obtidas para excluir qualquer derrame pericárdico de base. De volta ao AD, uma deflexão posterior com um torque suave no sentido horário permite uma visão longitudinal da veia cava superior (VCS). Essa visão permite a visualização adequada de cateteres provenientes de cavas (superior e inferior) e é também a visão padrão para monitorar o avanço das guias e bainhas transseptais ( figura 1 ).

Um longo fio-guia foi, então, inserido através da veia femoral direita e a progressão suave para o VCS foi confirmada pelo ICE. É possível visualizar o apêndice atrial direito perto do óstio da VCS, e o posicionamento inadvertido do fio-guia nessa estrutura pode, então, ser evitado. Uma longa bainha transeptal foi avançada sobre a guia e colocada na VCS (usando ICE, pode-se ver a bainha “cobrindo” o fio-guia, enquanto a parte distal do fio-guia permanece brilhante).

Quando os cateteres multipolares são inseridos através das veias femorais, usando bainhas curtas, é possível ver o avanço do cateter por mapeamento EA e pelo ICE, até que potenciais elétricos apareçam nos polos distais, confirmando a “chegada” no AD.

### Posicionamento dos cateteres

Quando o sistema CARTO foi utilizado, um cateter de ablação irrigado com sensor de força de contato foi avançado guiado pela visualização de EA e ICE, e um mapa atrial direito limitado foi construído, criando assim uma matriz (permitindo que os outros cateteres sejam visualizados em mapas EA), e delineando a anatomia septo e SC ( vídeo 1 ). Essa etapa não é necessária com o sistema NAVx, com o qual qualquer cateter pode ser visualizado sem necessidade de construção de matriz. O SC foi, então, canulado com o cateter multipolar, também sob visualização pelo mapa EA e ICE (O ICE mostra claramente a porção proximal do SC e seu óstio, assim como a entrada do cateter). A progressão desses cateteres multipolares foi monitorada e confirmada usando mapeamento EA (se o cateter vem da veia femoral, a anatomia da cava veia inferior é criada) ou pelo ICE (se o cateter vem da veia jugular interna, pode ser claramente avançando na VCS). Quando o cateter duodecapolar foi usado, os 10 polos distais foram colocados no SC e os polos proximais no AD. Um cateter quadripolar foi colocado no ventrículo direito usando a mesma técnica.

**Vídeo 1:**
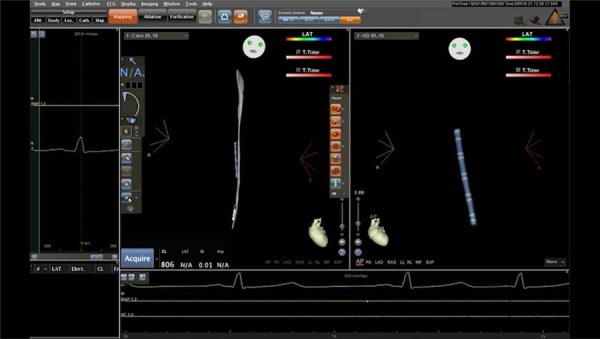
– Inserção de cateter a partir do acesso femoral ao AD guiada pelo sistema de mapeamento eletroanatômico. Após os cateteres chegarem no AD (cateter decapolar seguido do cateter de ablação [RF]), marcado pelo aparecimento de eletrogramas, a anatomia atrial é reconstruída e o seio coronário canulado – primeiro pelo cateter RF e em seguida pelo decapolar. Acesse o vídeo aqui: https://bit.ly/3gOojgU.

### Pacientes com FA

Nos casos de FA, uma vez criada uma geometria rápida do AD (sistema CARTO apenas), dois fios-guia foram inseridos na SVC e o posicionamento adequado confirmado pelo ICE. Duas longas bainhas transseptais (curva fixa e deflectível) foram avançadas sobre o fio até a VCS. É importante ressaltar que a heparina foi dada assim que os acessos venosos foram obtidos, antes de qualquer inserção de cateter, visando um TCA > 350s. Estes níveis eram mantidos até o final da instrumentação do átrio esquerdo através de infusão contínua de heparina e novos bolus, quando necessário.

Duas punções transseptais foram realizadas separadamente e guiadas continuamente pelo ICE, descendo até o septo a partir da VCS ( figura 1 e vídeo 2 ). Após cada perfuração septal, um fio-guia foi avançado para a veia pulmonar superior esquerda (PV), permitindo assim o posicionamento seguro das bainhas sobre a guia na cavidade atrial esquerda (LA). O cateter de ablação e um cateter de mapeamento multipolar foram então posicionados nas VPs. Todas essas etapas foram claramente visualizadas no ICE, que também poderia ser colocado na própria cavidade atrial esquerda através de um dos acessos transseptais para uma visualização de altíssima definição (feita nos últimos 15 casos desta série). Foi colocado também um cateter esofágico multipolar (para monitorar a temperatura durante aplicações de radiofrequência [RF]) e sua posição ajustada pela visualização no ICE.

**Vídeo 2:**
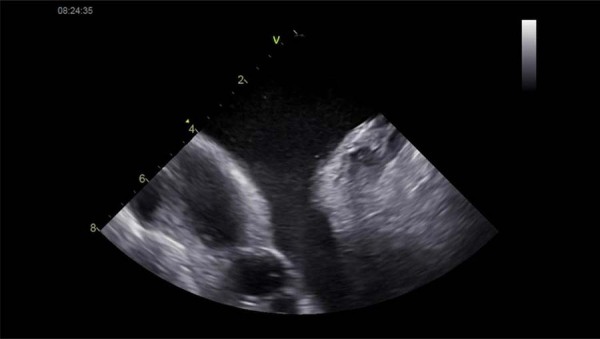
– *Punção transseptal “zero-fluoro”. Após o posicionamento do ICE no AD, o AE e a VCS são visualizados. O fio-guia chega a VCS seguido de avanço da bainha sobre a guia. A posição da bainha na SVC é confirmada por injeção salina mostrando fluxo craniocaudal de bolhas. A tenda septal e a perfuração são mostradas, seguidas do avanço da guia para as VPs esquerdas. O posicionamento da bainha é então confirmado na cavidade atrial esquerda também por visualização de bolhas com injeção de solução salina. A segunda bainha transseptal é então recuada da VCS para o septo, seguindo-se, então, uma segunda punção transseptal.* Acesse o vídeo aqui: https://bit.ly/3gOojgU.

**Figura 2:**
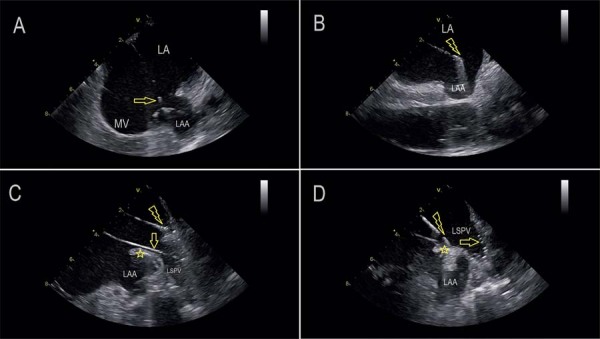
– Sequência de imagens do ICE durante mapeamento e ablação no átrio esquerdo. Estas imagens foram obtidas após o cateter ICE ser colocado na cavidade atrial esquerda (LA) através do septo. A) Um cateter de mapeamento multipolar de alta densidade (Pentarray – Biosense Webster, marcado por seta) está coletando dados anatômicos e elétricos ao redor do apêndice atrial esquerdo (LAA). MV – válvula mitral. B) A ponta do cateter de ablação com sensor de força de contato está flutuando na cavidade atrial esquerda. Como não está tocando em nenhuma estrutura, este é um bom local para calibrar o sensor como força zero. Este passo é necessário antes de começar as aplicações de RF. LAA – apêndice atrial esquerdo. C) O cateter de ablação é destacado no teto do átrio esquerdo (LA) em torno da veia pulmonar superior esquerda (LSPV). O cateter de mapeamento (seta) está dentro da veia pulmonar (LSPV) monitorando sua atividade elétrica e a conexão com o átrio esquerdo. É nítido que o cateter de ablação está no antro da veia pulmonar e não aplicando energia no seu interior. Asterisco marca a prega entre a veia superior esquerda e o apêndice atrial (LAA). D) Ablação na prega (*) entre a veia superior esquerda e o apêndice atrial (LAA). O cateter de mapeamento está dentro da veia pulmonar (LSPV – seta).

**Figura 3:**
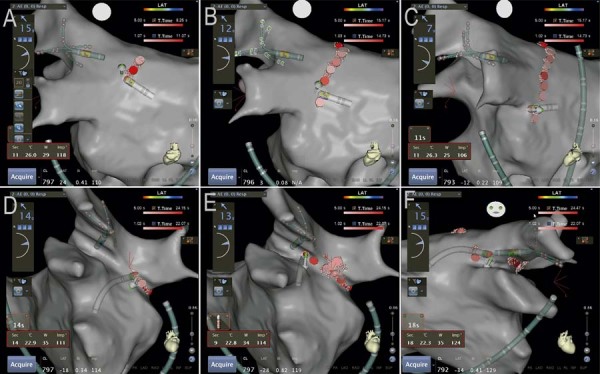
– *Sequência de imagens durante a ablação circunferencial ao redor das veias pulmonares esquerdas para isolamento. São mostradas imagens 3D guiadas pelo sistema CARTO e as marcações das aplicações de RF (pontos rosas e vermelhos) ao redor das veias esquerdas. Observa-se que o cateter de ablação fornece informações de força de contato, a seta representando o vetor de força e no canto superior esquerdo o número de gramas quantificando o contato com o tecido (entre 7 e 15g neste exemplo); pontos mais escuros significam mais contato com o tecido e tempo de aplicação de energia. Também é mostrado um cateter de mapeamento multipolar na veia superior esquerda (LSPV – Pentarray – Biosense Webster), para monitorar sua atividade elétrica e confirmar o isolamento.*

A anatomia do átrio esquerdo e veias pulmonares foi reconstruída por um mapa de alta densidade usando o cateter multipolar ( vídeo 3 ). Em particular, a prega entre a PV superior esquerda e o apêndice atrial esquerdo foi visualizada no ICE (com o cateter colocado no ventrículo direito ou na própria cavidade atrial esquerda – figura 2 ) e sua posição anotada manualmente no mapa EA. Após a calibração do sensor de contato, foi realizado isolamento circunferencial ponto a ponto para ambos os pares de veias pulmonares ( figura 3 e vídeo 4 ), utilizando 40W de potência máxima de RF e força de contato entre 10-20g. Na presença de aumento da temperatura do esôfago, que ocorre nos segmentos posteriores, foram utilizadas aplicações de RF mais curtas (5-10 segundos) e/ou menor potência (25-30W). Após o isolamento, infusões de Adenosina (18 mg) foram utilizadas para confirmar a ausência de condução residual.

**Vídeo 3:**
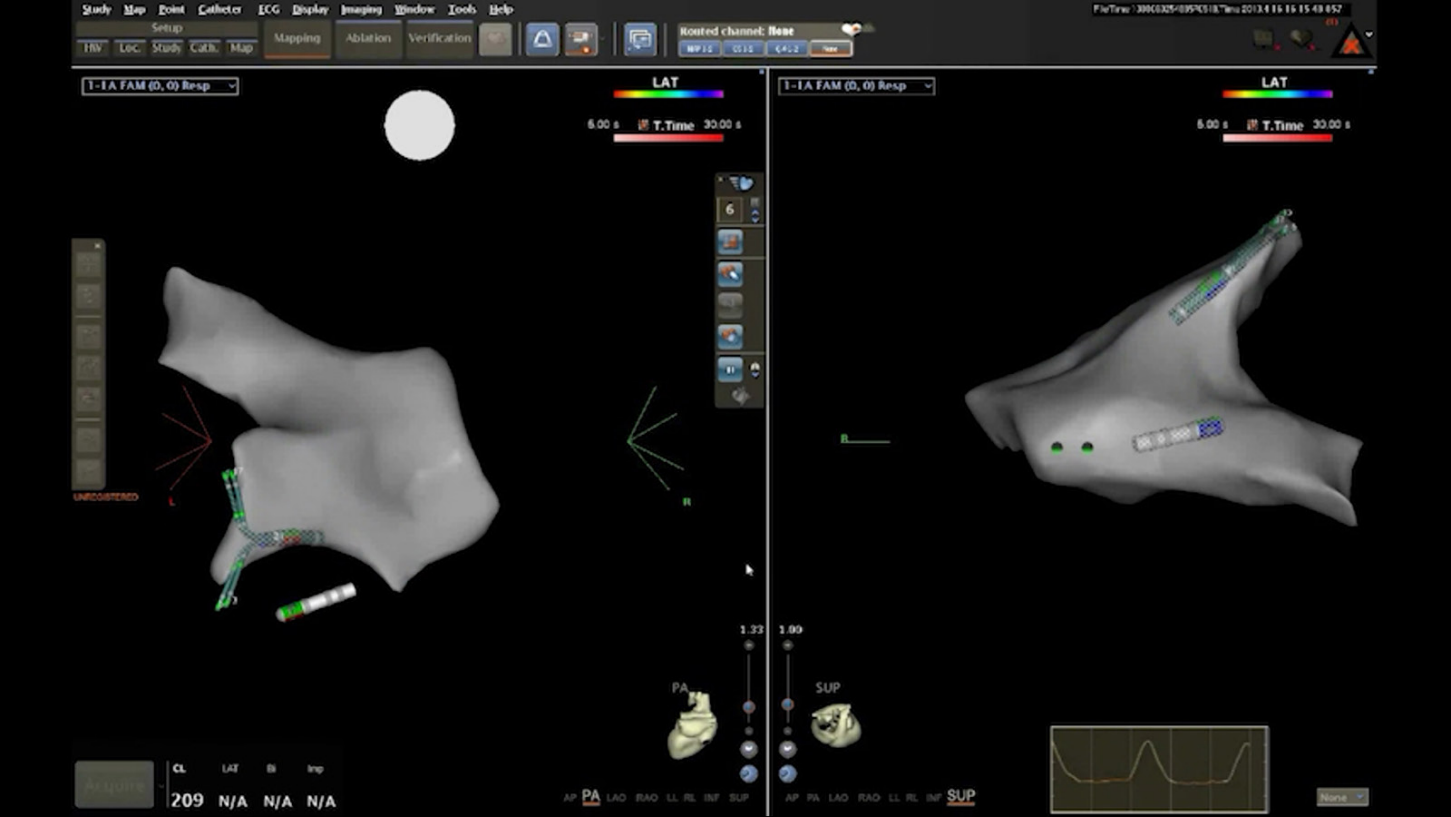
– *Reconstrução anatômica de alta densidade da cavidade atrial esquerda e das VPs. A aquisição anatômica é obtida movendo, sequencialmente, o cateter de mapeamento multipolar, enquanto o cateter de ablação está estacionado no anel mitral. Duas visões diferentes são mostradas (posterior e superior).* Acesse o vídeo aqui: https://bit.ly/3gOojgU.

**Vídeo 4:**
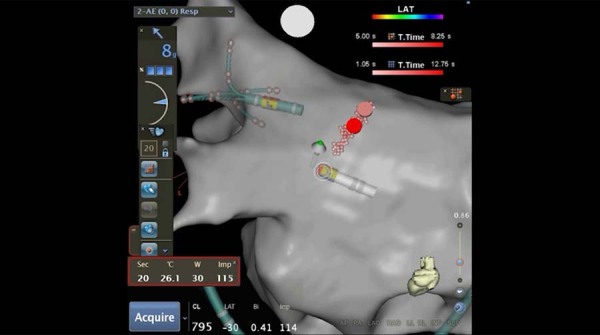
– *Imagens 3D guiadas pelo sistema CARTO demonstrando as aplicações de RF (pontos rosas e vermelhos) ao redor das VPs esquerdas. O cateter de ablação fornece informações de força de contato – a seta mostra o vetor de força e no canto superior esquerdo o número de gramas quantificando o contato tecidual. Sítios de aplicações de RF cobrindo toda a circunferência ao redor das VPs esquerdas são visualizados.* Acesse o vídeo aqui: https://bit.ly/3gOojgU.

Infusão de alta dose de Isoproterenol a uma taxa de 20 mcg/10 min foi rotineiramente realizada em busca de focos extrapulmonares indutíveis, que foram mapeados e ablacionados quando presentes. Em pacientes com flutter atrial típico documentado, o cateter de ablação foi trazido de volta ao RA, e uma lesão linear guiada pelo ICE no istmo cavo-tricuspídeo foi realizada. A visualização detalhada ao ICE foi essencial para evitar embaraçar o cateter com os eletrodos de marcapasso, quando presentes. Em anatomias desafiadoras (por exemplo, proeminente válvula de Eustáquio ou na presença de depressões), o ICE é fundamental para garantir o contato adequado com o tecido ao longo da linha.

Recuperar o acesso transseptal ao LA, sempre que necessário, foi fácil usando os mesmos sítios de acesso previamente marcados no mapa EA. Durante o procedimento, por segurança, o cateter ICE foi frequentemente prolapsado no ventrículo direito para verificar se havia derrame pericárdico, nos seguintes tempos: (1) no início do procedimento, (2) após as punções transseptais, (3) após o isolamento das veias esquerdas, (4) após o isolamento das veias direitas e (5) ao final do procedimento. O ICE permite também a imediata detecção de trombos, que não são detectados por nenhum outro método de imagem não baseado em ultrassom.

Em pacientes com marcapasso, a avaliação do dispositivo foi realizada antes e depois do procedimento para garantir a integridade dos eletrodos.

O fechamento do acesso às veias femorais foi feito com suturas em “figura de oito” com Prolene “0” para atingir a hemostasia total. Protamina era usada na dose máxima de 50 mg IV para reversão parcial da anticoagulação. A deambulação foi permitida após 6h, e a anticoagulação oral foi retomada no mesmo dia.

### Pacientes com SVT

Para os casos de SVTs, foi utilizada uma rotina semelhante aos casos de FA. Para facilitar o avanço do cateter multipolar na ausência de bainhas transseptais, foram preferidas bainhas longas que já entregam os cateteres na cava inferior, evitando assim as tortuosidades anatômicas dos vasos femorais e ilíacos.

**Figura 4:**
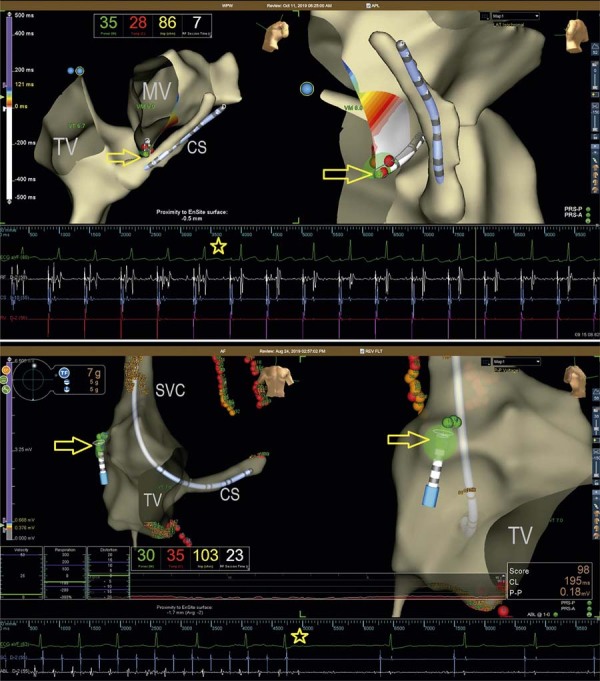
– Mapeamento e ablação para taquicardia supraventricular. A ablação de uma via acessória (WPW) no anel mitral é mostrada no painel superior, onde o cateter de ablação (seta) é posicionado na porção septal do anel. A aplicação de RF leva à eliminação imediata da condução pela via e à normalização do QRS (*). No painel inferior, uma taquicardia atrial foi mapeada e ablacionada no átrio direito (seta), com interrupção da arritmia (*) e retorno ao ritmo sinusal normal. MV – válvula mitral. TV – válvula tricúspide. SVC – veia cava superior. CS – seio coronário.

A partir desse local, a progressão para o AD foi marcada pelo aparecimento de eletrogramas atriais e visualização pelo ICE, como descrito. Marcos anatômicos como o feixe de His e SC, veias cavas e apêndice atrial direito foram marcados nos mapas EA, sob orientação do ICE ( figura 4 ).

### População estudada

Esta série reporta pacientes consecutivos, não selecionados, encaminhados para procedimentos de ablação por cateter para o tratamento de arritmias atriais (FA, flutter e SVTs) realizados sem uso de fluoroscopia, guiados exclusivamente pelo mapeamento eletroanatômico e ICE. O software Excel (versão 2019) foi utilizado para tabulação dos dados. Os principais objetivos são descrever a viabilidade desta estratégia inovadora e demonstrar seu perfil de segurança.

**Figura 5:**
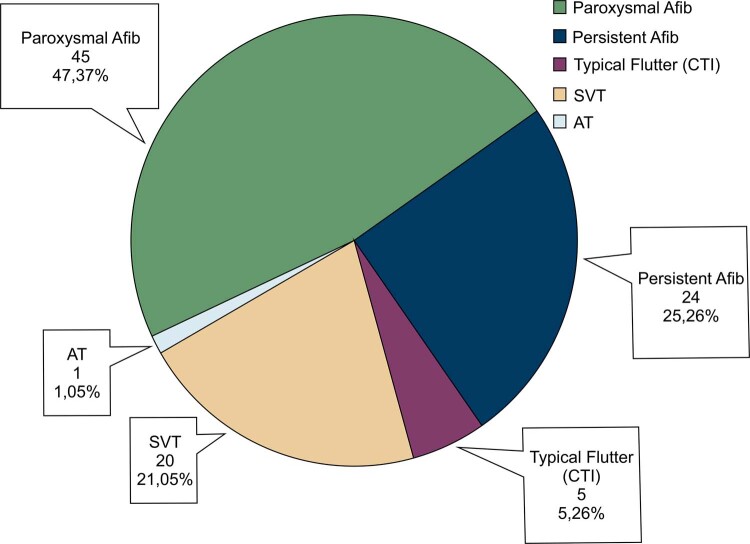
– *Distribuição dos pacientes segundo o tipo de arritmia. AT – taquicardia atrial; CTI – flutter típico no istmo cavo-tricuspídeo; SVT – taquicardia supraventricular (reentrada nodal AV ou WPW); Afib – fibrilação atrial.*

De maio/2019 a dezembro/2019, 95 pacientes (idade média de 60 ± 18 anos, 61% do sexo masculino) foram submetidos à abordagem “zero fluoro”, com a seguinte distribuição de procedimentos: ablação de FA (69 pts [73%], 45 FA paroxística [47%] e 24 FA persistente [25%]) ou SVT (26 pts [27%) – 14 reentrada nodal [15%], 6 WPW [6% - 4 no anel mitral e 2 no anel tricúspide), 5 flutter atrial direito típicos [5%], 1 taquicardia atrial [1%]). Em pts com FA, a média do volume atrial esquerdo era 36 ± 4 ml/m^2^ , e 36% (25 pts) apresentavam cardiopatia estrutural, incluindo doenças valvares reumáticas (3 pts – 3%) e funcionais (8pts – 8%), doença coronária (17 pts – 17%) e pós-cirurgia cardíaca (12 pts – 12%, que apresentam cicatrizes e suturas no AD, AE e septo). Os pacientes e as características dos procedimentos estão resumidos na tabela 1 e na figura 5 .

**Tabela 1:** – Características dos pacientes

Característica	N = 95
Idade (anos)	60 ± 18
Sexo masculino	58 (61%)
Carto	62 (65%)
Navx	33 (34%)
Índice de massa corporal	22,5 ± 2,8
Hipertensão	71 (75%)
Diabetes Mellitus	48 (51%)
Doença isquêmica do coração	31 (33)%

O protocolo incluía internação hospitalar de 24h em uma unidade com telemetria. Nenhum exame de imagem era necessariamente realizado antes da alta hospitalar. Tomografias computadorizadas não foram realizadas antes e nem no seguimento, enquanto outros métodos de imagem sem radiação foram utilizados a critério do médico responsável.

## Resultados

Não foram observados derrames pericárdicos, complicações trombóticas ou outras complicações nesta série. Todos os alvos de ablação em ambos os átrios foram alcançados e adequadamente visualizados. Todas as aplicações planejadas foram realizadas, o que significa que a falta de imagens fluoroscópicas não impediu a entrega de RF a nenhum sítio. Esses locais incluíram o antro das VPs, parede posterior do AE, parede anterior, septo, apêndice atrial esquerdo, apêndice atrial direito, SC, istmo cavo-tricuspídeo, anel mitral e tricúspide, via lenta nodal e crista terminalis (AD). Não foi usado fluoroscopia de backup, e nenhum avental de chumbo foi necessário em qualquer paciente.

Punções transseptais difíceis (devido à fossa oval pequena, septo elástico ou fibroso), observado em 19 pacientes (20%), foram administradas sem uso de fluoroscopia em todos os casos. Este é um achado significativo, uma vez que há uma crença comum de que a visualização fluoroscópica transseptal de todo o conjunto bainha-agulha é essencial tanto para a perfuração septal, a penetração no AE e a troca de bainhas sobre guias. Todos esses passos foram claramente visualizados usando o ICE de forma otimizada. O mesmo se aplica à passagem por ramos venosos tortuosos para avançar o cateter ICE – todos os casos foram gerenciados com sucesso sem fluoroscopia por visualização cuidadosa do espaço livre de eco e/ou inserção de fios-guia.

Eletrodos de marcapasso definitivo estiveram presentes em 9 pacientes (9,5%), 7 marcapassos de dupla câmara (DDD) e 2 dispositivos desfibriladores-ressincronizadores com 3 eletrodos (AD, VD e SC). Cinco pacientes (56%) eram dependentes da estimulação devido a bloqueio AV completo sem qualquer ritmo de escape. Em 3 desses casos, foram realizados mapeamento e ablação no AD (istmo cavo-tricuspídeo e flutter relacionado à cicatriz de atriotomia), além de instrumentação do AE e isolamento das VPs. Todos esses casos também foram devidamente concluídos sem fluoroscopia. É importante ressaltar que as avaliações após o procedimento não mostraram nenhum dano, desposicionamento ou alterações de limiar dos eletrodos. Importante salientar que se deve ter cuidado para diferenciar fios-guia dos eletrodos permanentes no ICE.

## Discussão

Essa série de casos destaca a viabilidade, a segurança e a eficácia de uma abordagem “zero- fluoro” para tratar tanto FA quanto diferentes tipos de arritmias atriais, mesmo na presença de eletrodos de marcapasso (mesmo em pacientes dependentes). Nesse caso, é de extrema importância que o mapeamento EA e o ICE sejam usados de forma ótima.^17 - 21^

Nossa série representa uma experiência pioneira no Brasil e na América Latina usando uma abordagem livre de radiação. Resultou de uma preocupação antiga sobre a necessidade de redução da radiação e da implementação constante de etapas independentes de raios-X ao nosso protocolo de ablação. Já tínhamos experiência significativa no uso do ICE e mapeamento EA em todos os casos de FA, nos últimos 16 anos, o que certamente facilitou nossa curva de aprendizado. Nesse sentido, não foi observado aumento de custo em nossa série, pois exatamente os mesmos cateteres são utilizados nos procedimentos utilizando fluoroscopia.

A capacidade de usar o mapa EA e o ICE para fornecer visualização adequada de cada etapa do procedimento já foi relatada. Razminia et al.,^22^ compararam, retrospectivamente, a segurança e a eficácia entre dois grupos (60 procedimentos de ablação não fluoroscópica e 60 fluoroscópicos). Não foi observado aumento significativo de complicações ou no tempo de procedimento, com eficácia comparável. O grupo fluoroscópico teve uma exposição média de raios-X de 33 minutos em casos de ablação de FA. Bulava et al.,^14^ relataram estudo com 80 pacientes randomizados para serem submetidos a isolamento elétrico das veias pulmonares guiado por fluoroscopia ou utilizando apenas ICE e CARTO 3 com cateter de força de contato. Não foi encontrada diferença na sobrevida livre de arritmias após 12 meses. Não foram registradas complicações graves em ambos os grupos. Nessa série, o grupo fluoroscópico teve uma exposição média de 3 min para ablações de FA, mostrando que os operadores já tinham experiência no uso de imagens não fluoroscópicas. Juntos, esses dados sugerem que a adoção de medidas de redução de radiação pode afetar drasticamente a exposição a raios-X mesmo em procedimentos fluoroscopicamente guiados, sem prejuízos na segurança.

O mapeamento EA é ferramenta fundamental no procedimento, uma vez que fornece uma geometria confiável para orientar a navegação e aplicações de RF, mas pode potencialmente fornecer informações enganosas se não for rigorosamente usada. A descrição inicial de Reddy et al.,^12^ relataram uma série de 20 procedimentos consecutivos de ablação de FA sem o uso de fluoroscopia, contando apenas com imagens ICE e o sistema NAVx para criar geometria. Nessa série, a integração da imagem EA com uma tomografia computadorizada de átrio esquerdo adquirida previamente foi utilizada na maioria dos pacientes, exigindo acesso à artéria femoral e mapeamento da raiz aórtica para criar uma fusão confiável das imagens. Novas tecnologias, como cateteres de mapeamento com múltiplos polos de registro e softwares de reconstrução, podem gerar uma geometria com menos trauma, de forma mais rápida e confiável. Esses sistemas permitem um mapa de alta densidade com melhor delineamento de anatomia, comparável a uma reconstrução de tomografia computadorizada, sem a necessidade de exposição à radiação ou acesso arterial. Em nossa série, nenhum paciente foi submetido à tomografia pré-ablação. Além disso, os sistemas EA fornecem orientação da movimentação de cateter permitindo movimentos facilmente reprodutíveis e uma excelente correlação entre torque, deflexão e força de contato.

Em nosso país, apenas duas empresas fornecem sistemas de mapeamento EA – sistema CARTO 3 (Biosense Webster, Diamond Bar, CA, EUA) e sistema Ensite-NavX (St. Jude Medical, St. Paul, MN, USA). Quando esses dois sistemas foram comparados para mapeamento e ablação (4), os resultados (sucesso agudo, complicações e taxas de recorrência) foram semelhantes. Em nosso estudo, o CARTO 3 foi utilizado em 67,8% dos pacientes e o sistema NavX, em 32,2% de todos os procedimentos ( Tabela 1 ), com resultados semelhantes.

A visualização do ICE é fundamental em cada passo de uma ablação complexa não fluoroscópica. Com a varredura completa do ICE, todas as etapas podem ser adequadamente monitoradas, até mesmo quando os cateteres saem pela ponta da bainha (certificando-se que não pressione a parede atrial). Nenhum passo é cego usando essa abordagem, mesmo ao avançar cateteres ou guias no sistema venoso até o coração. A visualização e a canulação do SC são melhores do que com fluoroscopia. Sem mencionar as punções transseptais, que são, indubitavelmente, melhor visualizadas no ICE. Baykaner et al.,^23^ relatou, recentemente, 747 punções transseptais sem uso de fluoroscopia, realizadas em 646 pacientes, em 5 centros nos EUA, usando diferentes abordagens para alcançar a fossa oval. O acesso transseptal foi associado a uma baixa taxa de complicações (0,7%). Em nosso estudo, foram realizadas 142 punções transseptais sem qualquer complicação. É fato que uma curva de aprendizado relativamente curta é necessária para se tornar confortável e proficiente na manipulação do ICE. Mas, definitivamente, dá informações melhores e mais detalhadas do que a fluoroscopia.

Razminia et al.,^15^ relataram 5 anos de acompanhamento de ablações sem fluoroscopia em uma série de 500 pacientes. Os procedimentos foram realizados de forma segura e eficaz, com taxas semelhantes de recorrência e complicações quando comparadas com a técnica padrão. Em nossa série, também não observamos nenhuma complicação significativa. À medida que essa técnica se torna a prática padrão para procedimentos ainda mais complexos, como taquicardias ventriculares, um aumento na taxa de complicações poderia ser esperado. Por isso, relatos da segurança e eficácia para os pacientes são extremamente importantes e, associado a um treinamento mais difundido de ICE e mapeamento EA, serão vitais para a adoção em larga escala desses procedimentos na prática clínica.

Todas as ferramentas necessárias para uma ablação sem radiação já estão disponíveis na maioria dos laboratórios de eletrofisiologia e são familiares para a maioria dos eletrofisiologistas.^24^ O engajamento neste campo só precisa de uma equipe motivada com uma mudança de mentalidade. Uma vez feito, é um caminho sem volta. É altamente benéfico para os pacientes que se submetem, frequentemente, a mais de um procedimento ablação e usam outras modalidades diagnósticas ou terapêuticas que utilizam, ao longo de sua vida, radiação (por exemplo, tomografias, intervenções coronárias) e que, geralmente, ou não são contabilizadas ou são negligenciadas. O risco é cumulativo ao longo do tempo. Temos que ter isso em mente, especialmente, frente ao preocupante crescimento de cânceres, nas estatísticas, e levando em consideração que o impacto pode ocorrer anos após a exposição.

Intervenções sem radiação também permitem um tratamento seguro de pacientes grávidas. As diretrizes europeias mais recentemente publicadas pelo ESC, para o tratamento de arritmias supraventriculares,^25^ dão uma indicação **IIa** em centros experientes. Mesmo em casos convencionais de SVTs, onde procedimentos simples com sedação e uso de apenas 2 cateteres são frequentemente utilizados, há valor o uso de ICE e anestesia geral. Ambos contribuem para um procedimento seguro e confortável para pacientes e médicos e adicionam a possibilidade de induzir apneia transitória para melhorar a estabilidade dos cateteres em aplicações próximas ao nódulo AV / feixe de His.

A fluoroscopia zero também é altamente benéfica para a equipe de saúde. Em primeiro lugar, reduzir a exposição à radiação é obviamente desejado para pessoas que têm exposição diária por anos. É muito incômodo os relatos de aumento de até 1% no risco de câncer.^3 , 7^ São preocupantes os relatos mostrando que 85% dos cânceres cerebrais em médicos intervencionistas ocorrem no hemisfério esquerdo,^26 - 28^ sugerindo uma relação causal entre a exposição ocupacional e os efeitos da radiação (já que o lado esquerdo é sabidamente mais exposto do que o direito). Sem mencionar o considerável benefício de evitar o uso de aventais de chumbo pesados, o que, ao longo do tempo, torna as questões ortopédicas uma ocorrência quase unânime.^29 - 31^ Os autores não podem enfatizar, suficientemente, o alívio que horas de pé sem uso de aventais de chumbo representa.

“Zero-fluoro” é, então, altamente benéfico para os pacientes e toda a equipe de saúde. Múltiplas exposições à radiação são comuns, na era moderna, com as modalidades de imagem disponíveis. Normalmente, não percebemos a natureza cumulativa de múltiplas exposições e seus potenciais efeitos prejudiciais a longo prazo. Pacientes submetidos à ablação frequentemente tiveram ou terão exposição repetida à tomografia, à fluoroscopia, à angiografia coronária e periférica, bem como a exames nucleares. Um procedimento livre de radiação cujos custos, segurança e eficácia sejam, ao menos, semelhantes à alternativa padrão baseada em fluoroscopia, mesmo na presença de eletrodos permanentes, é, portanto, altamente valioso. Uma equipe motivada com uma mudança de mentalidade é fundamental nesse sentido. É nossa percepção que, após uma curva de aprendizado, na maioria dos casos, a visualização e a manipulação de cateteres são, de fato, mais precisas do que com a fluoroscopia, sem nenhuma parte cega.

## Limitações

Relatamos um número, relativamente, pequeno de pacientes sem um grupo controle. Os procedimentos de fluoroscopia zero foram realizados por operadores com grande experiência em mapeamento 3D e ICE, e a reprodutibilidade de nossos resultados por operadores menos experientes pode variar devido a uma curva de aprendizado mais prolongada. No entanto, acreditamos que esses resultados são significativos e representam a base para futuras avaliações sobre a segurança e a eficácia dessas técnicas.

## Conclusões

Uma estratégia de ablação por cateter sem radiação (“zero-fluoro”) para tratamento de FA e outras arritmias atriais é agudamente segura e eficaz quando guiada pela utilização adequada do ICE e do mapeamento 3D. Diversos sítios em ambos os átrios puderam ser alcançados e adequadamente ablacionados sem a necessidade de fluoroscopia de backup. Não foram observadas complicações.
