# Supplementary material for: Ablação Por Cateter Sem Uso de Fluoroscopia Para Tratamento de Fibrilação Atrial e Arritmias Atriais: Eficácia e Segurança
Source: Arq Bras Cardiol. 2020 Jun 29;114(6):1015–26. [Article in Portuguese] doi: 10.36660/abc.20200096 (PMC8416121; doi:10.36660/abc.20200096)
Supplement: Translation

# Translation: Catheter Ablation for Treatment of Atrial Fibrillation and Supraventricular Arrhythmias Without Fluoroscopy Use: Acute Efficacy and Safety

**Authors:** Eduardo B. Saad, Charles Slater, Luiz Antonio Oliveira Inácio, Gustavo Vignoli dos Santos, Lucas Carvalho Dias, Luiz Eduardo Montenegro Camanho

**Affiliations:** 1 Brazil Hospital Pró-Cardíaco - Serviço de Arritmias e Estimulação Cardíaca Artificial, Rio de Janeiro, RJ - Brazil

**Keywords:** Arrhythmias, Cardiac, Atrial, Fibrillation, Catheter Ablation, Fluoroscopy, radiation, Efficacy, Safety

## Abstract

**Background:**

The use of ionizing radiation in medical procedures is associated with significant health risks for patients and the health care team.

**Objectives:**

Evaluate the safety and acute efficacy of ablation for atrial fibrillation (AF) and supraventricular arrhythmias (SVTs) using an exclusively non-fluoroscopic approach guided by intracardiac echo (ICE) and 3D-mapping.

**Methods:**

95 pts (mean age 60 ± 18 years, 61% male) scheduled for AF Ablation (69 pts, 45 paroxysmal AF and 24 persistent AF) or non-AF SVT (26 pts – 14 AV node reentry, 6 WPW, 5 right atrial (RA) flutters, 1 atrial tachycardia) underwent zero fluoro procedures. Nine patients (9.5%) had permanent pacemakers or defibrillator resynchronization (CRT-D) devices. Both CARTO (65%) and NAVx (35%) mapping systems were used, as well as Acunav and ViewFlex ICE catheters.

**Results:**

Pulmonary vein isolation (PVI), as well as all other targets that needed ablation in both atria were reached and adequately visualized. No pericardial effusions, thrombotic complications or other difficulties were seen in these series. Difficult transseptal puncture (19 patients - 20%) was managed without fluoroscopy in all cases. No backup fluoroscopy was used, and no lead apparel was needed. Pacemaker interrogations after the procedure did not show any lead damage, dislocation, or threshold changes.

**Conclusions:**

A radiation-free (fluoroless) catheter ablation strategy for AF and other atrial arrhythmias is acutely safe and effective when guided by adequate ICE and 3D-mapping utilization. Multiple different bi-atrial sites were reached and adequately ablated without the need for backup fluoroscopy. No complications were seen. (Arq Bras Cardiol. 2020; 114(6):1015-1026)

## Introduction

Catheter ablation is currently the most effective treatment for atrial fibrillation AF,^1 , 2^ Atrial flutter, and supraventricular tachycardias (SVTs). It is widely performed in various centers around the world, giving the increasing prevalence of AF in the population and the modest response to anti-arrhythmic medications.

As with most percutaneous cardiac procedures, fluoroscopy has been a primary imaging modality to manipulate catheters in the vascular space and cardiac chambers. However, ionizing radiation has multiple potential deleterious effects for both patients and the healthcare team.^3 - 6^ These effects are cumulative, and all of us are continuously exposed nowadays due to high usage in diagnostic and therapeutic imaging modalities.^7^

In that regard, the **ALARA** ( **A** s **L** ow **A** s **R** easonably **A** chievable) principle has been proposed to minimize radiation use to the minimum needed to meet the objective.^3^ In recent years, several efforts have succeeded in reducing radiation exposure during catheter ablation procedures, including a reduction in fluoroscopy times and doses,^8 , 9^ better shielding, and especially regarding other non-fluoroscopic imaging modalities – namely 3D electroanatomical systems (EA) and intracardiac echocardiography (ICE).

Those fluoroscopy reduction tools have been increasingly used in the electrophysiology (EP) lab over the years, so that it became possible to guide the entire ablation procedure and thus avoid the use of X-ray^10^ entirely. First reported about 10 years ago,^11 - 13^ those Zero-Fluoro techniques are gaining popularity in the EP community, as they are as safe and as effective as the ones guided by fluoroscopy.^14 - 16^

## Objectives

The purpose of this study was to demonstrate the feasibility and safety of catheter ablation of atrial fibrillation, atrial flutter, and supraventricular tachycardias without the use of fluoroscopy, using exclusively electroanatomic mapping and intracardiac echocardiography in a series of 95 consecutive patients in a single center.

## Methods

### Description of the technique

All procedures were performed under general anesthesia, and venous access was obtained using ultrasound guidance, according to the specific need of the procedure, but generally consisted of two or three right femoral vein punctures, one left femoral vein puncture (for the ICE catheter), and one internal jugular vein puncture (in atrial fibrillation cases, for coronary sinus duodecapolar catheter placing). Monitoring during the procedure included 12-lead electrocardiogram and EA mapping cutaneous patches (Ensite NavX - St. Jude Medical, St. Paul, MN, USA or CARTO 3 – Biosense Webster Inc., Diamond Bar, CA, USA).

### Navigation through the intravascular space

From the left femoral vein, an ICE catheter (ViewFlex Xtra – Abbott or Acunav – Biosense Webster) was advanced to the right atrium (RA), guided by visualization of echo-free spaces in the vascular system.

The ICE catheter was advanced through the left iliac vein while keeping an “echo-free space” close to the near-field of the ultrasound image (representing an absence of tissue contact at the tip of the ICE catheter). This technique allows the operator to discriminate between a free-advancement of the ICE catheter tip through the vascular lumen when echo-free space is visible and a palpable resistance to advancement when this image pattern is not obtained.

Whenever the path to the inferior vena cava was not so clear, a retained guidewire technique, using long wires from the left femoral vein allowed advancing the ICE catheter following a clear wire image in the lumen. That is of particular value in patients with thin iliac veins, where echo-free space is not very clear.

Upon reaching the inferior vena cava, it is possible to identify the cavoatrial junction where, at the level of the liver, and a parenchymatous image with clearly visible intrahepatic veins is appreciated. At this moment, it is essential to identify and avoid the inadvertent progression of the ICE catheter through the hepatic veins and correctly direct it to the right atrium (RA), which can be done with gentle anterior deflection. Once in the RA, all standard views can be obtained using the conventional technique of clockwise or counterclockwise torque from the home view. The catheter is then prolapsed to the right ventricle using anterior deflection, while keeping the tricuspid valve visible, and sectional views of the pericardial space were obtained to rule out any baseline pericardial effusion. Back to the RA, a posterior deflection with a gentle clockwise torque allows a longitudinal view of the superior vena cava (SVC). This view allows adequate visualization of catheters coming from superior veins and is the standard view to start advancing transseptal guidewires and sheaths ( figure 1 ).

**Figure 1:** – ICE imaging sequence showing the steps for the zero-fluoro double transseptal puncture. A) A guidewire (arrow) is advanced to the superior vena cava (SVC); in the picture, the right atrium (RA) is also visualized, as well as the right atrial appendage (*), confirming correct wire positioning. B) A long transseptal sheath (arrow) is advanced over the wire to the SVC, erasing the brightness of the wire as it is advanced. C) The transseptal sheath + needle assembly (arrow) in the SVC, to be pulled down to the fossa ovalis. The left atrium (LA) is visualized, as well as a transseptal access that has been previously performed. D) Sheath + needle (arrow) pulldown along the septum on its way to the fossa ovalis. E) Sheath + needle tenting the fossa ovalis (arrow), confirming adequate positioning to provide access to the LA. F) Puncture of the fossa ovalis (FO) and needle enhancement visualized in the LA cavity (arrow). The transseptal puncture is performed in a posterior location, confirmed by visualization of the left inferior PV (LIPV) in the ultrasound plane.

A long guidewire was then inserted through the right femoral vein, and smooth progression to the SVC was confirmed by ICE imaging. It is possible to visualize the right atrial appendage near the SVC ostium, and the inadvertent misplacement of the guidewire in this structure can then be avoided. A long transeptal sheath was advanced over the wire and placed in the SVC (using ICE, one can see the sheath itself “covering” the guidewire, while the distal, unsheathed part of the guidewire remains bright).

When multipolar catheters are inserted through the femoral veins using short sheaths, it is possible to see catheter advancement using EA mapping and ICE image, until electrical potentials appear in the distal poles, confirming “arrival” at the RA.

### Catheter positioning

When the CARTO system was used, an irrigated ablation catheter with contact force sensor was then advanced under EA and ICE visualization and a limited right atrial map was constructed, mostly to create a matrix (allowing other catheters to be visualized in EA maps) and to delineate the septum and coronary sinus (CS) anatomy ( video 1 ). This step is not needed when the NAVx system is used, where any catheter can be visualized without the need for matrix creation. The CS was then cannulated with the multipolar catheter, under EA and ICE visualization (ICE can clearly visualize the proximal CS and the ostium, as well as the catheter going in). The progression of these multipolar catheters was monitored and confirmed using EA mapping (if the catheter is coming from the femoral vein, the inferior vena cava geometry is created) or ICE imaging (if the catheter is coming from the internal jugular vein, it can be clearly seen coming from the SVC). If a duodecapolar catheter was used, the distal 10 poles were placed in the CS and the proximal poles in the RA. A quadripolar catheter was placed in the RV using the same technique.

**Video 1:** – *Catheter insertion from the femoral access to the RA guided by the electroanatomic mapping system. After the catheters arrive in the RA (decapolar catheter followed by the ablation catheter [RF]), marked by the appearance of electrograms, the RA anatomy is created, followed by cannulation of the coronary sinus (CS) – first by the RF catheter and followed by the decapolar one.* Access the video here: https://bit.ly/2XWhIbE.

### AF Cases

In AF cases, once a limited RA geometry was created (CARTO system only), two guidewires were inserted from the right femoral vein and advanced to the SVC, with adequate positioning being visualized on ICE. Two long transseptal sheaths (fixed curve and deflectable) were advanced over the wire to the SVC. Importantly, heparin was given as soon peripheral access was obtained, before any catheter insertion, aiming at an activated clotting time (ACT) > 350s. Those levels were maintained until left atrial instrumentation ended by continuous infusion and rebolus as needed.

Two transseptal punctures were separately performed under ICE visualization, coming down to the septum from the SVC ( figure 1 and video 2 ). After each septal perforation, a guidewire was advanced to the left superior pulmonary vein (PV), thus allowing for safe passage over the wire sheath positioning in the left atrial (LA) cavity. The ablation catheter and a multipolar mapping catheter were then positioned in the PVs. All these steps were clearly visualized on ICE, which could also be placed in the LA cavity through one of the transseptal accesses for a very high definition visualization (done in the last 15 cases of these series). A multipolar esophageal catheter was placed, and its position guided by ICE visualization.

**Video 2:** – *Zero-fluoro transseptal puncture. After the ICE catheter is positioned in the RA, the LA and SVC are visualized. The guidewire arrives in the SVC, followed by sheath advancement. Sheath position in the SVC is confirmed by saline injection, showing craniocaudal flow. Septal tenting and perforation are shown, followed by wire advancement to the left PV. The sheath is then confirmed in the LA cavity by saline bubble visualization. The second transseptal sheath is then pulled down from the SVC to the septum, followed by a second septal perforation.* Access the video here: https://bit.ly/2XWhIbE.

The LA and PV anatomies were reconstructed with a high-density map using the multipolar catheter ( video 3 ). In particular, the ridge between the left superior PV and left atrial appendage was visualized on ICE (with the catheter placed in the right ventricle or in the LA cavity itself – figure 2 ) and its position manually annotated in the EA map. After calibrating the contact sensor, point-by-point circumferential PV isolation was performed for both pairs of veins ( figure 3 and video 4 ), using 40W of maximal power and contact force between 10-20g. Whenever esophageal temperature rose in the posterior segments, shorter (5-10 seconds) RF applications and/or lower power (25-30W) were used. Adenosine challenge (18 mg) was used to confirmed PV isolation without dormant conduction.

**Video 3:** – *High definition anatomic reconstruction of LA and PVs. With the multipolar mapping catheter, the anatomic acquisition is obtained by sequentially moving the mapping catheter, while the ablation catheter is parked in the mitral annulus. Two different views are shown (posterior and superior).* Access the video here: https://bit.ly/2XWhIbE.

**Figure 2:** – ICE imaging sequence examples of LA mapping and ablation. These were recorded after the ICE catheter was placed in the LA cavity across the septum. A) A multipolar high-density mapping catheter (Pentarray – Biosense Webster, marked by arrow) is collecting anatomic and electric data around the left atrial appendage (LAA). MV – mitral valve. B) The tip of the contact force-sensor ablation catheter is floating in the LA cavity. As it is not touching any structure, this is a good spot to calibrate the sensor as zero force. This step is needed before initiating RF delivery. C) The ablation catheter is highlighted at the roof of the LA around the LSPV. The mapping catheter (arrow) is inside the LSPV monitoring its electrical connection to the LA. It is clear that the ablation catheter is in the PV antrum and not delivering energy inside the vein. D) Ablation in the ridge (*) between the LSPV and the LAA. The mapping catheter is inside the LSPV (arrow).

**Figure 3:** – *Sequence of imagens during circumferential ablation around the left PVs for isolation. Shown are the CARTO-guided 3D images of ablation lesions (pink and red dots) placed around the left PVs. Note that the ablation catheter provides contact-force information, the arrow depicting the force vector and on top-left, the number of grams quantifying the tissue contact (between 7 and 15g in this example), darker dots meaning more tissue contact and energy delivery. Also shown is a multipolar mapping catheter in the LSPV (Pentarray – Biosense Webster) to monitor its electrical activity and confirm isolation.*

**Video 4:** – *CARTO-guided 3D images of RF delivery (pink and red dots) placed around the left PVs. The ablation catheter provides contact-force information, the arrow showing the force vector, and on top-left, the number of grams quantifying the tissue contact. Ablation lesions covering the full circumference around the left PVs are shown.* Access the video here: https://bit.ly/2XWhIbE.

High-dose Isoproterenol infusion at a rate of 20 mcg/10 min was performed in search of inducible extra-pulmonary foci, which were ablated when present. In patients with documented typical atrial flutter, the ablation catheter was then pulled to the RA, and a linear, ICE guided lesion was performed in the cavotricuspid isthmus (CTI). Detailed ICE visualization was essential to avoid tangling the catheter with pacemaker leads, when present. In challenging anatomies (e.g., prominent Eustachian ridge or the presence of pouches), ICE is critical to ensure adequate tissue contact throughout the CTI.

Regaining access to the LA, whenever needed, was easily accomplished using previously tagged transeptal access sites in the EA map. During the procedure, to ensure safety, the ICE catheter was frequently prolapsed to the right ventricle to check for pericardial effusion, at the following times: (1) at baseline, (2) after transeptal punctures, (3) after left PV isolation, (4) after right PV isolation, (5) and at the end of procedure. ICE also allows for immediate detection of clot formation, which cannot be seen with other non-ultrasound imaging modalities.

In pacemaker patients, device interrogation was performed before and after the procedure to guarantee lead integrity.

Femoral vein access care was performed with figure-of-eight sutures with Prolene “0” to achieve full hemostasis. Protamine at a maximal dose of 50 mg IV was given to allow for partial reversal of anticoagulation. Deambulation was allowed after 6h, and oral anticoagulation was resumed on the same day.

### SVT Cases

For SVT cases, a routine somewhat similar to AF cases was used. To facilitate multipolar catheter advancement in the absence of transseptal sheaths, long sheaths that deliver the catheters in the inferior vena cava (IVC) were preferred, thus avoiding the anatomical tortuosity of the femoral and iliac vessels.

Starting from that site, progression to the RA was marked by the appearance of atrial electrograms and ICE visualization, as described. Anatomical landmarks such as His bundle and CS, SVC, IVC, and right atrial appendage ostia were tagged in the EA maps under ICE guidance ( figure 4 ).

**Figure 4:** – Mapping and ablation for supraventricular tachycardia. Ablation of an accessory pathway (WPW) in the mitral annulus is shown in the upper panel, where the ablation catheter (arrow) is positioned in the septal part of the annulus. RF application leads to the immediate elimination of conduction and normalization of the QRS (*). In the lower panel, an atrial tachycardia was mapped and ablated in the RA (arrow), with interruption of the arrhythmia (*) and return to normal sinus rhythm. MV – mitral valve. TV – tricuspid valve. SVC – superior vena cava. CS – coronary sinus.

### Population studied

We report a series of consecutive, unselected cases of catheter ablation procedures for the treatment of atrial tachyarrhythmias (AF, atrial flutter, and SVTs) performed without fluoroscopy, exclusively guided by ICE and EA mapping. Excel software (2019 version) was used for data tabulation. The main goals are to describe the feasibility of this innovative approach and to show the safety profile of this technique.

From May/2019 to December/2019, 95 consecutive patients (mean age 60 ± 18 years, 61% males) referred for ablation underwent the zero fluoro approach, with the following procedure distribution: AF Ablation (69 pts [73%], 45 paroxysmal AF [47%] and 24 persistent AF [25%]) or non-AF SVT (26 pts [27%] – 14 AV node reentry [15%], 6 WPW syndrome [6% - 4 in the mitral and 2 in the tricuspid annulus], 5 right atrial (RA) typical flutters [5%], 1 atrial tachycardia [1%]). In AF pts, the mean LA volume was 36 ± 4 ml/m^2^ and 36% (25 pts) had structural heart disease, including rheumatic (3 pts - 3%) and other types of valvular disease (8 pts - 8%), coronary artery disease (17 pts - 17%), post-open heart surgery pts (12 pts - 12%, which usually have LA, RA and septal scars and sutures). Patients and procedural characteristics are summarized in table 1 and figure 5 .

**Table 1:** – Patients’ characteristics

Characteristics	N = 95
Age (years)	60 ± 18
Male gender	58 (61%)
Carto system	62 (65%)
Navx system	33 (35%)
Body Mass Index (BMI)	22.5 ± 2.8
Arterial Hypertension	71 (75%)
Diabetes Mellitus	48 (51%)
Ischemic Heart Disease	31 (33%)

**Figure 5:** – *Distribution of patients according to the type of arrhythmia. AT – atrial tachycardia; CTI – cavotricuspid isthmus. SVT – supraventricular tachycardia (AV node reentry or WPW).*

The protocol included an overnight hospital stay in a telemetry bed. No routine imaging was required before hospital discharge. CT scans were performed neither at baseline nor in the follow-up, while other radiation-free imaging methods were used at the discretion of the follow-up physician.

## Results

No pericardial effusions, thrombotic complications, or other difficulties were seen in these series. All targets in both atria that needed ablation were reached and adequately visualized. All intended ablations were performed, meaning that the lack of fluoroscopic imaging did not hamper RF delivery. Those sites included the PV antra, LA posterior wall, anterior wall, septum, LA appendage, RA appendage, CS, CTI, mitral and tricuspid annulus, slow pathway, and crista terminalis. No backup fluoro was used, and no lead apparel was needed in any patient.

Interestingly, difficult transseptal puncture (due to small fossa, floppy septum, or fibrous septum), which occurred in 19 patients (20%), was managed without fluoroscopy use in all cases. This is a significant finding, since there is a common belief that transseptal fluoroscopic visualization of the entire sheath-needle assembly is essential both for septal perforation, penetration, and sheath over the wire exchange. All these steps were clearly visualized using ICE to its best advantage. The same applied to the negotiation of tortuous venous branches to advance the ICE catheter – all cases were successfully managed without fluoroscopy by careful visualization of the echo-free space and guidewire insertion.

Permanent pacemaker leads were present in 9 patients (9,5%), 7 dual chamber (DDD) pacemakers, and 2 CRT-D devices with 3 leads (RA, RV, and CS leads). Five patients (56%) were pacemaker-dependent due to complete AV block without any escape rhythm. In 3 of these cases, RA mapping and ablation (CTI and atriotomy scar-related flutters) were performed on top of LA instrumentation and PV isolation. All these cases were also adequately completed without fluoroscopy. Importantly, device interrogations after the procedure did not show any lead damage, dislocation, or threshold changes. Of note, care must be taken to differentiate the guidewire from lead imaging on ICE.

## Discussion

This series of cases highlights the feasibility, safety, and efficacy of a zero fluoro approach when treating both AF and different types of atrial arrhythmias, even in the presence of pacemaker leads (and even in pacer-dependent patients). For that matter, it is of utmost importance that ICE, and EA mapping be used to their best advantage.^17 - 21^

Our series represents a pioneer experience in Brazil and Latin America using a radiation-free approach. It resulted from a long-lasting concern about radiation reduction and steady implementation of non-X-ray steps to our ablation protocol. We already had significant expertise from using ICE and EA in every AF case for the last 16 years, which surely made our learning curve easier. In that regard, no increase in costs was seen in our series, as precisely the same catheters were used as in the procedures using fluoroscopy.

The ability to use EAM and ICE to provide adequate visualization of every step of the procedure has already been reported. Razminia et al.^22^ retrospectively compared safety and efficacy between two groups (60 non-fluoroscopic and 60 fluoroscopic ablation procedures). No significant increase in complications or procedure time was observed, with comparable efficacy. The fluoroscopic group had an average X-ray exposure of 33 minutes in AF ablation cases. Bulava et al.^14^ reported on 80 patients randomized to either fluoroscopically-guided PVI or PVI without fluoroscopy using ICE and the CARTO 3 system with contact force ablation catheters. No difference in arrhythmia-free 12-month survival was found. No severe complications were recorded in either group. In this series, the fluoroscopy group had an average exposure of 3 min for AF ablations, showing that the operators were already experts in the use of non-fluoroscopic imaging. Taken together, these data suggest that the adoption of radiation reduction measures can dramatically affect x-ray exposure even in fluoroscopically-guided procedures, with no safety concerns.

EA mapping is a fundamental part of the procedure since it provides a reliable geometry to guide the roving catheter and RF applications but could potentially provide misleading information if not stringently used. The initial description by Reddy et al.^12^ reported a series of 20 consecutive AF ablation procedures without the use of fluoroscopy, relying only on ICE images and the NavX EA system to create geometry. In this series, EA image integration with a previously acquired left atrium CT scan was used in the majority of patients, requiring femoral artery access and aortic root mapping to create a reliable fusion between aortic anatomy from EA mapping and CT image. New technologies, such as multielectrode mapping catheters and software can provide a less traumatic, fast, and reliable geometry, with a high-density map and better anatomy delineation, comparable to a CT scan reconstruction, without the need to expose the patient to radiation and avoiding arterial access during the procedure. In our series, no patient was submitted to pre-ablation CT scans. Also, the EA systems provide ablation catheter tip color-coding orientation that allows easily reproducible movements and an excellent correlation between torque, deflection, and contact force.

In our country, only two companies currently provide EA mapping systems - Carto 3 system (Biosense Webster, Diamond Bar, CA, USA) and Ensite-NavX system (St. Jude Medical, St. Paul, MN, USA). When these two systems were compared for mapping and CTI catheter ablation, Macias et al. showed^4^ that the results (acute success, complications, and recurrence rates) from both EA mapping systems were similar. In our study, Carto 3 was used in 67.8% of patients, and the NavX system in 32.2% of all procedures ( Table 1 ), with similar results.

ICE visualization is critical in every step of a non-fluoroscopic complex ablation. With thorough ICE scanning, all the steps can be adequately monitored, even when catheters come out of the sheath tip (making sure it does not force the atrial wall). There is no blind step using this approach, even when advancing catheters or wires in the venous system to the heart. CS visualization and cannulation are better than with fluoroscopy, not to mention the transseptal punctures, which are undoubtedly best visualized on ICE. Baykaner et al.^23^ recently reported on 747 zero-fluoroscopy transeptal punctures, performed in 646 patients in 5 different centers across the US, using different approaches to reach the fossa ovalis. The transseptal access was associated with a low total complication rate (0.7%). In our study, a total of 142 transseptal punctures were performed with no complications. Indeed, a somewhat short learning curve is needed to become comfortable with and proficient in ICE manipulation. But it definitively gives better and more detailed information than fluoroscopy.

Razminia et al.^15^ reported a 5-year follow-up of fluoroless ablations in a series of 500 patients. These procedures were safely and effectively performed, with similar rates of recurrence and complications when compared with the standard technique. In our series, we also did not observe any significant complications. As this technique becomes the standard practice for even more complex procedures, such as ventricular tachycardias, a rise in complications rate could be expected. As such, reports on the safety and effectiveness for the patient are extremely important and will, together with more widespread training in ICE and EA mapping, be vital to large-scale adoption of these procedures in clinical practice.

All the tools needed for a successful radiation-free ablation are already available in most EP labs and familiar to most EP physicians.^24^ Engagement in this field only needs a motivated team with a change in mindset. Once one does it, there is no way back. It is highly beneficial to patients – who can frequently undergo more than one ablation, usually have other diagnostic or therapeutic modalities that use radiation (e.g., CT scans, coronary interventions) over their lifespan, which are usually unaccounted for or neglected. The risk is cumulative over time. We have to keep that in mind, especially when cancer statistics show a worrying steep rise and when the impact can occur years after exposure.

Radiation-free interventions also allow safe ablation treatment of pregnant patients. The most recently published ESC Guidelines for the treatment of supraventricular arrhythmias^25^ gives an **IIa** indication in experienced centers. Even for standard SVT cases, where simple procedures under conscious sedation and using two catheters are frequently performed, it is worthwhile using ICE and general anesthesia. They allow for safe and comfortable procedures for both pts and physicians and add the possibility of using transient apnea to enhance catheter stability when dealing with arrhythmias near the AV node / His bundle.

Zero fluoroscopy is also highly beneficial to the health care team. First, reducing the radiation exposure is obviously desired for people who have been exposed daily for years. It is a matter of concern to read reports of an increase of up to 1% in one’s lifetime risk of cancer;^3 , 7^ it is uncomfortable to read reports that 85% of brain cancers in interventional physicians occur on the left hemisphere,^26 - 28^ suggesting a causal relation to occupational exposure to radiation effects (since the left side is known to be more exposed than the right). Not to mention the considerable benefit of avoiding using heavy lead aprons, which, over time, makes orthopedic issues an almost unanimous occurrence.^29 - 31^ The authors cannot stress enough the massive relief that standing hours without having to wear heavy lead aprons represents.

It is also highly beneficial to patients and all the healthcare team. Multiple exposures to radiation are common in the modern era, with the readily available imaging modalities. We usually do not realize the cumulative nature of multiple exposures and their potential detrimental effects over the long term. Patients undergoing ablation not uncommonly have had or will have repeated exposure to CT, fluoroscopy, coronary and peripheral angiography, as well as nuclear scans. A radiation-free procedure with at least similar costs, safety, and effectiveness as the standard fluoro-based alternative, even when additional hardware is implanted in the heart, is thus highly valuable. A motivated team with a mindset change is critical in that regard. It is our perception that, after a learning curve, in most instances, the visualization and manipulation are indeed more precise than that with fluoroscopy, without any blind parts.

## Limitations

We report on a relatively small number of patients and lack a control group. Zero fluoroscopy procedures were performed by operators with a large experience in ICE and 3D mapping, and the reproducibility of our results by less experienced operators may vary due to the need for a steeper learning curve. However, we believe that these results are meaningful and represent the basis for further evaluations about the safety and efficacy of these techniques.

## Conclusions

A radiation-free (fluoroless) catheter ablation strategy for AF and other atrial arrhythmias is acutely safe and effective when guided by adequate ICE and 3D mapping utilization. Multiple different bi-atrial sites could be reached and adequately ablated without the need for backup fluoroscopy. No complications were seen.
